# Bioactive Hydrogels for Spinal Cord Injury Repair: Emphasis on Gelatin and Its Derivatives

**DOI:** 10.3390/gels11070497

**Published:** 2025-06-26

**Authors:** Alexandra Daniela Rotaru-Zavaleanu, Marius Bica, Sorin-Nicolae Dinescu, Mihai Andrei Ruscu, Ramona Constantina Vasile, Andrei Calin Zavate, Venera Cristina Dinescu

**Affiliations:** 1 Department of Epidemiology, University of Medicine and Pharmacy of Craiova, 2-4 Petru Rares Str., 200349 Craiova, Romania; alexandra.rotaru@umfcv.ro (A.D.R.-Z.); sorin.dinescu@umfcv.ro (S.-N.D.); ruscumihai@gmail.com (M.A.R.); 2 Experimental Research Centre for Normal and Pathological Aging, University of Medicine and Pharmacy of Craiova, 200349 Craiova, Romania; 3 Department of Surgery, University of Medicine and Pharmacy of Craiova, 2-4 Petru Rares Str., 200349 Craiova, Romania; marius.bica@umfcv.ro; 4 Department of Pediatric Surgery, University of Medicine and Pharmacy of Craiova, 2-4 Petru Rares Str., 200349 Craiova, Romania; 5 Department of Health Promotion and Occupational Medicine, University of Medicine and Pharmacy of Craiova, 2-4 Petru Rares Str., 200349 Craiova, Romania; venera.dinescu@umfcv.ro

**Keywords:** biopolymer gels, hydrogels, particle synthesis, biomedical applications, chitosan hydrogels, hyaluronic acid hydrogels, alginate hydrogels, silk fibroin hydrogels, gelatin hydrogels, GelMA, SCI repair

## Abstract

Spinal cord injuries (SCIs) present a major clinical challenge, often resulting in permanent loss of function and limited treatment options. Traditional approaches, including surgery, drugs, and rehabilitation, have had modest success in restoring neural connectivity due to the complex pathophysiology of SCI. In recent years, bioactive hydrogels have gained attention as a versatile platform for neural repair. Their ability to mimic the extracellular matrix, deliver therapeutic agents, and support cell survival makes them promising tools in regenerative medicine. This narrative review highlights the latest advances in hydrogel-based therapies for SCI, with a focus on innovations such as self-healing, conductive, and anti-inflammatory hydrogels. We also explore hybrid approaches that integrate nanomaterials, stem cells, and bioelectronics to address both primary and secondary injury mechanisms. While various hydrogel systems have been investigated, we place particular emphasis on gelatin-based hydrogels, especially gelatin methacryloyl (GelMA), due to their emerging clinical relevance. GelMA stands out for its bioactivity, tunable mechanics, and compatibility with 3D printing, making it a strong candidate for personalized therapies and scalable production. Unlike previous reviews that broadly summarize hydrogel use, this work specifically contextualizes gelatin-based platforms within the wider landscape of SCI repair, underscoring their translational potential. We also address current challenges, such as immune response, long-term integration, and clinical validation, and suggest future directions for bridging the gap from bench to bedside.

## 1. Background

SCIs remain among the most devastating medical conditions, significantly affecting the physical, psychological, and social well-being of individuals globally with an estimated annual incidence of between 250,000 and 500,000 cases. SCI imposes an immense socioeconomic burden, particularly due to its association with lifelong disability [1]. Despite advances in acute care and rehabilitation, the inherent complexity of spinal cord structure and function presents significant challenges to achieving meaningful recovery, and traditional treatment strategies, such as surgical stabilization, pharmacological interventions, and physical rehabilitation, have had limited success in restoring neural connectivity and long-term functional independence [2,3].

The pathophysiology of SCI is classically divided into two interrelated phases. The primary injury occurs immediately following the traumatic event, typically involving mechanical forces such as compression, contusion, or laceration. This acute trauma leads to vascular disruption; compromise of the blood–spinal cord barrier; and immediate necrotic and apoptotic cell death, affecting not only neurons but also glial and endothelial cells. Damage to the nerve fiber tracts further disrupts spinal cord architecture and connectivity. This initial insult sets the stage for a complex and prolonged secondary injury phase, which unfolds over hours to weeks post-injury. Triggered by the primary damage, this phase involves a cascade of pathological processes including hemorrhage, ionic imbalance, excitotoxicity, oxidative stress, and immune cell infiltration. These events drive neuroinflammation, demyelination, astrogliosis, and extracellular matrix (ECM) remodeling, collectively worsening the initial lesion and creating an inhibitory environment for regeneration. The interplay between these mechanisms not only amplifies short-term tissue destruction but also impairs long-term functional recovery by limiting axonal regrowth and neuroplasticity [4].

In this context of extremely complex physiopathology, bioactive hydrogels have emerged as a promising tool in the field of regenerative medicine and tissue engineering [5]. Hydrogels are hydrophobic polymeric networks with a high water content that closely mimic the ECM, providing a favorable environment for cell survival, proliferation, and differentiation, and their biocompatibility, tunable mechanical properties, and ability to incorporate bioactive agents make them particularly suitable for neural tissue engineering applications when properly designed [6]. Bioactive hydrogels can serve as platforms for drug delivery and gene therapies, as supports for cell transplantation, and also as vehicles for the controlled release of growth factors, thus becoming extremely versatile in the repair of spinal cord injuries, both in their acute and chronic phases [7].

The potential of bioactive hydrogels is constantly increasing due to advances in materials science that have led to the creation of multifunctional hydrogels that have improved properties such as self-repair, conductivity, and anti-inflammatory action by combining peptides, nanoparticles, and functional biomolecules [8]. In this way, not only do transplanted cells remain away from hostile microenvironments but they also influence the SCI microenvironment to aid in remyelination and axonal regeneration [9]. For example, electrically conductive hydrogels have been shown to aid neuronal signaling, while hydrogels with controlled degradation profiles ensure that the scaffold continues to provide support throughout the healing process [10]. Bioactive hydrogels have the ability to combine physical, chemical, and biological signals, which positions them as a central element in the development of next-generation therapies for SCI [7]. They also have an excellent structure for combination therapies that integrate multiple strategies, such as stem cell transplantation, drug delivery, and electrical stimulation, to achieve synergistic effects in promoting neuronal repair, while their injectability and bio-adhesion properties facilitate minimally invasive administration, a crucial advantage for translation into clinical practice when it comes to SCI [11].

In recent years, among these bioactive hydrogels, gelatin-based hydrogels, including unmodified gelatin, GelMA, and interpenetrating polymer networks (IPNs) containing gelatin, have taken a central role due to their excellent biocompatibility, enzymatic degradability, and structural similarity to native ECMdG G. Historically derived from collagen, gelatin has long been recognized for its cell-adhesive motifs and ease of modification. Innovations such as GelMA, which allows tunable crosslinking via photopolymerization, and hybrid gelatin-based systems combining synthetic and natural polymers, have significantly advanced their applicability in neural tissue engineering, drug delivery, and scaffold design for SCI repair. These materials offer a dynamic platform for minimally invasive delivery, biofunctional signaling, and integration with electrical or biochemical therapies, reinforcing their position as a cornerstone in the development of next-generation SCI treatments [12,13].

Despite all the promising progress, there are still many challenges, and the most important one is related to the design of hydrogels that can perfectly integrate with the host tissues and maintain functionality in the long term. This type of hydrogel requires careful analysis of their mechanical, chemical, and biological properties. An equally important challenge is the lack of full translation into human clinical trials to verify the efficacy of these biomaterials, thus highlighting the need for further research and development. This review aims to provide an overview of the latest advances in the use of bioactive hydrogels for SCI repair, examining the design and synthesis of these materials, how they can be used in specific biomedical applications, and how they play a role in solving the problems associated with SCI treatment.

### Materials and Methods

The literature search was conducted using the PubMed database to identify relevant studies focused on the application of polymers and hydrogels, particularly gelatin-based systems, in SCI repair. The search strategy included combinations of keywords such as “polymers”, “spinal cord injuries”, “gelatin”, and “hydrogels”, employing Boolean operators (e.g., AND, OR) to refine the query and capture a comprehensive body of literature. The final search string used was (spinal cord injuries [MeSH Terms] OR spinal cord injury OR SCI) AND (polymer OR biopolymer OR synthetic polymer) AND (gelatin OR gelatin-based) AND (hydrogel OR hydrogel-based).

Only articles that were available as free full texts were included in the analysis to ensure accessibility and replicability of findings. Additional inclusion criteria were that publications be written in English and present original experimental data or systematic reviews related to the design, synthesis, or biomedical application of hydrogel systems in SCI models. Editorials, commentaries, and non-peer-reviewed content were excluded. The initial search yielded 95 free full-text articles. Following independent screening and eligibility assessment by two reviewers, 24 articles were deemed relevant and included in the final analysis.

This approach allowed for a focused evaluation of recent advances in bioactive hydrogel technologies and their therapeutic potential in neural tissue engineering for SCI.

## 2. Multifaceted Approaches to Spinal Cord Injury Repair: Physical, Chemical, and Biological Strategies

### 2.1. Physical Methods

A key basis for SCI repair is represented by physical methods that use external stimuli to enhance cellular and tissue regeneration, and of these, electrical stimulation has attracted significant attention due to its ability to enhance neural activity and promote axonal regeneration [14]. Functional electrical stimulation (FES) is frequently combined with rehabilitation protocols to enhance motor function recovery. It delivers low-intensity electrical impulses that activate neural circuits, thereby promoting synaptic connectivity and reducing muscle atrophy [15].

**Figure 1:**
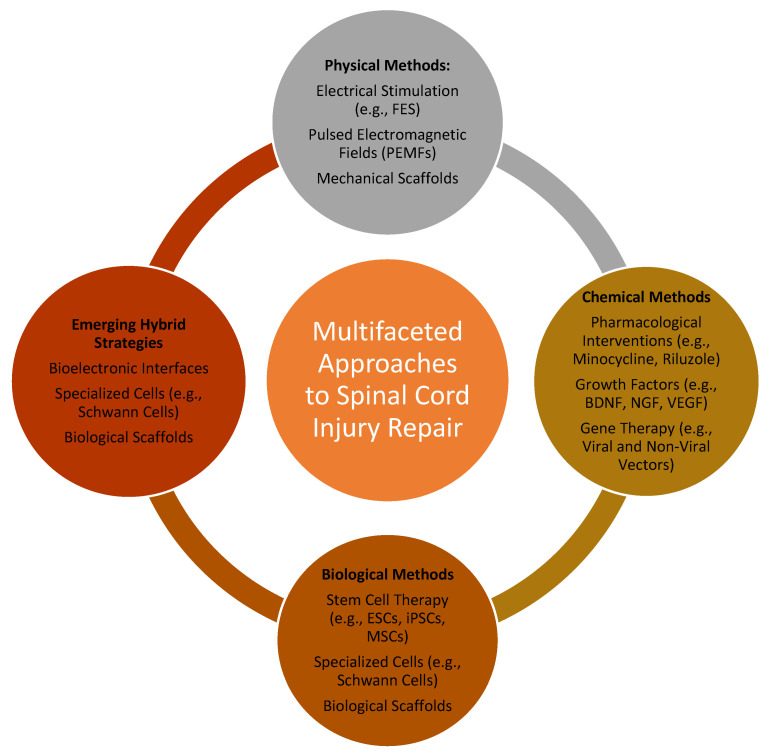
Integrated strategies for spinal cord injury repair: a multifaceted approach. Comprehensive depiction of physical, chemical, biological, and hybrid strategies for spinal cord injury repair, highlighting the synergy of advanced methodologies (FES—functional electrical stimulation; PEMFs—pulsed electromagnetic fields; BDNF—brain-derived neurotrophic factor; NGF—nerve growth factor; VEGF—vascular endothelial growth factor; ESCs—embryonic stem cells; iPSCs—induced pluripotent stem cells; MSCs—mesenchymal stem cells).

Another promising approach is the use of pulsed electromagnetic fields (PEMF), which are effective in modulating cellular behavior, reducing inflammation, and improving the overall microenvironment for regeneration, while mechanical stimulation strategies, such as those using biomimetic scaffolds with aligned fibers, provide a physical framework for cellular guidance and axonal regeneration, mimicking the natural architecture of the spinal cord (Figure 1) [16].

### 2.2. Chemical Methods

Pharmacologic interventions, usually represented by anti-inflammatory drugs and antioxidants, are chemical strategies targeting the biochemical microenvironment of the spinal cord to attenuate secondary injury and promote repair, thus playing a crucial role in minimizing the damage caused by inflammation and oxidative stress. Minocycline, an anti-inflammatory agent, has demonstrated its potential in preserving neuronal integrity, while calcium channel blockers, such as riluzole, act by reducing excitotoxicity, a major factor in neuronal death after SCI [17].

An essential component of chemical methods, beyond simple molecules, is the delivery of growth factors, such as brain-derived neurotrophic factor (BDNF), nerve growth factor (NGF), and vascular endothelial growth factor (VEGF). These factors promote neuronal survival, axonal growth, and angiogenesis, creating a regenerative environment within the injured spinal cord, but their therapeutic efficacy largely depends on controlled and localized delivery systems [18].

Gene therapy complements chemical methods by targeting specific pathways involved in SCI pathology and is mostly represented by viral and non-viral vectors that can be used to deliver genes that stimulate the production of neuroprotective proteins or to reduce the expression of inhibitory molecules, such as chondroitin sulfate proteoglycans (CSPGs), known to prevent axonal regeneration [19].

### 2.3. Biological Methods

Biological approaches leverage the regenerative capabilities of living cells and biological molecules to repair and restore function in the injured spinal cord. Stem cell therapy has emerged as a powerful tool in this domain, with various cell types showing potential for SCI repair. Embryonic stem cells (ESCs) and induced pluripotent stem cells (iPSCs) are prized for their pluripotency, allowing them to differentiate into diverse neuronal and glial cell types. Meanwhile, mesenchymal stem cells (MSCs) and neural stem cells (NSCs) are valued for their ability to secrete trophic factors that enhance the survival of native cells and promote repair.

In addition to stem cells, specialized cells such as Schwann cells and oligodendrocyte precursor cells have been used to enhance remyelination and facilitate axonal regeneration. These cells play a crucial role in restoring the insulating myelin sheath around damaged axons, a key step in functional recovery. To support the integration and function of transplanted cells, biological scaffolds made from natural biopolymers like collagen or decellularized tissues provide a conducive environment for cell attachment, proliferation, and differentiation [20].

### 2.4. Emerging Hybrid Strategies and Advanced Technologies

The complexity of SCI necessitates hybrid strategies that integrate physical, chemical, and biological approaches for synergistic effects. Bioelectronic interfaces, which combine conductive hydrogels with electronic devices, are an exciting advancement. These interfaces not only provide real-time monitoring of neural activity but also deliver targeted electrical stimulation to enhance neuronal connectivity [21].

Combination therapies are also gaining traction, where stem cell transplantation is paired with drug delivery systems or electrical stimulation to address multiple aspects of SCI repair simultaneously. For instance, hydrogels loaded with stem cells and growth factors can provide a dual therapeutic effect by delivering cells and promoting their survival in the hostile SCI environment [22]. Furthermore, nanotechnology plays a critical role in these hybrid strategies. Nanoparticles and nanofibers allow precise delivery of therapeutic agents while enhancing the structural and functional properties of scaffolds [23].

The integration of advanced technologies like biofabrication and three-dimensional (3D) printing is also reshaping the landscape of SCI repair. These techniques enable the creation of customized scaffolds that replicate the intricate architecture of the spinal cord, providing unprecedented precision and effectiveness in tissue engineering [24].

## 3. Expanding the Role of Biopolymer Gels in Particle Synthesis and Biomedical Applications

Bioactive hydrogels derived from biopolymers offer exceptional platforms for synthesizing and incorporating functional particles, providing innovative solutions in advanced biomedical applications [25]. Their intrinsic properties, such as biocompatibility, tunable biodegradation, and versatile chemical functionalities, make them ideal candidates for particle-assisted therapeutic strategies. These hydrogels facilitate controlled synthesis, encapsulation, and delivery of particles for diverse biomedical purposes [26]. Among these, chitosan-based hydrogels stand out for their inherent antimicrobial properties, biocompatibility, and ability to form gels under mild conditions. Their cationic nature facilitates interactions with negatively charged molecules, enabling the encapsulation of nanoparticles and drugs. However, chitosan has limited solubility at physiological pH, and its mechanical properties are relatively weak, requiring reinforcement with other polymers. Chitosan-based hydrogels are typically produced through ionic gelation using agents like sodium tripolyphosphate (TPP) [27]. For particle synthesis, they enable the in situ reduction of silver nanoparticles (AgNPs), imparting antimicrobial properties for wound healing and infection control [28]. Li et al. developed chitosan-based nanoparticles functionalized with folic acid to enhance bioenzyme activity for spinal cord injury treatment. Folic acid was conjugated to the chitosan nanoparticles to target folate receptors, which are overexpressed in injured spinal cord tissues. The functionalized nanoparticles exhibited enhanced bioenzyme activity, promoting neural tissue regeneration and reducing inflammation in spinal cord injury models. This study highlights the potential of folic-acid-functionalized chitosan nanoparticles as a therapeutic strategy for spinal cord injury repair [29]. Wang et al. developed a chitosan-based scaffold infused with neurotrophin-3 (NT-3) to promote neural regeneration following spinal cord injury. The incorporation of NT-3 aimed to enhance neuronal survival and differentiation. In vivo experiments demonstrated that the NT3-chitosan scaffold facilitated the reconstruction of neural circuits and improved motor function recovery in adult rat models of spinal cord injury [30].

Hyaluronic acid (HA) is another widely used biopolymer, appreciated for its biocompatibility, role in inflammation modulation, and ability to promote cell adhesion and proliferation [31]. HA-based hydrogels are extensively utilized in neural tissue engineering and cancer therapy, where their ability to support cell infiltration and targeted delivery is crucial. Despite these advantages, HA hydrogels often suffer from limited mechanical strength and rapid degradation, which can limit their long-term functionality [32]. HA hydrogels are commonly crosslinked using covalent methods, such as aldehyde-modified HA reacting with hydrazides, or ionic gelation techniques. For particle synthesis, HA has been used to fabricate hybrid hydrogels containing gold nanoparticles (AuNPs) via in situ reduction, enabling photothermal therapy for neural and cancer treatments [33]. Elkhenany et al. developed a composite scaffold combining hyaluronic acid and polypyrrole-coated fibers to support human neural precursor cells (hNPCs) for spinal cord injury repair. Hyaluronic acid provides a hydrophilic environment conducive to cell survival and differentiation, while polypyrrole, a conductive polymer, was used to coat fibers within the scaffold, enhancing electrical conductivity essential for neural tissue engineering. Curcumin, known for its anti-inflammatory and antioxidant properties, was incorporated to modulate the injury microenvironment. In vivo experiments demonstrated that this multifunctional scaffold promoted neural tissue regeneration and improved motor function recovery in spinal cord injury models, highlighting its therapeutic potential [34].

Alginate-based hydrogels, derived from brown algae, are valued in SCI applications for their biocompatibility, mild gelation conditions, and efficient encapsulation capabilities. Their ability to form hydrogels via ionic crosslinking with calcium ions enables straightforward incorporation of therapeutic agents, particularly for sustained drug release in anti-inflammatory and neuroprotective contexts [35]. While native alginate lacks intrinsic cell-adhesive motifs, recent studies have focused on composite strategies to overcome this limitation by blending alginate with bioactive or conductive materials. For instance, Saadinam et al. engineered an injectable hydrogel by ionically crosslinking alginate with chitosan, enhancing both cell adhesion and structural support. This hybrid scaffold promoted neural tissue regeneration and improved motor outcomes in vivo, demonstrating its efficacy in acute SCI models [36]. In another example, Manzari-Tavakoli et al. developed a multifunctional scaffold combining alginate, polypyrrole, and nanochitosan to support placenta-derived stem cells. Polypyrrole imparted electrical conductivity critical for neuronal activity, while nanochitosan reinforced mechanical integrity and bioactivity. The scaffold was further functionalized with noggin, a neural differentiation factor, and successfully restored motor function in preclinical models [37]. These examples underscore the growing trend toward alginate-based hybrid hydrogels, where blending with complementary polymers and bioactive agents significantly extends their therapeutic utility in neural repair.

Silk fibroin, a protein derived from silkworm cocoons, offers excellent mechanical properties, biocompatibility, and slow degradation, making it suitable for scaffolding and neural tissue engineering. It supports cell attachment and proliferation, enhancing its applicability in regenerative medicine [38]. However, silk fibroin hydrogels often require complex processing and chemical modifications to achieve desired mechanical and biological properties. Silk fibroin hydrogels are typically produced through self-assembly processes, enhanced by enzymatic or covalent crosslinking. These hydrogels can be functionalized with graphene oxide (GO) or reduced graphene oxide (rGO), significantly improving their conductivity and mechanical strength for neural tissue engineering and spinal cord regeneration [39]. Feng et al. developed a hybrid nanofiber gel combining a designer peptide with silk fibroin to promote neural regeneration. The designer peptide was engineered to self-assemble with silk fibroin, forming a nanofiber gel that mimics the extracellular matrix. In vivo experiments demonstrated that this hybrid gel supported neural cell adhesion and growth, leading to improved functional recovery in spinal cord injury models. This study highlights the potential of combining designer peptides with natural proteins like silk fibroin to create effective scaffolds for neural tissue engineering [40]. Deng et al. developed a scaffold combining collagen and silk fibroin to support human umbilical cord-mesenchymal stem cells (hUC-MSCs) for spinal cord injury repair. Collagen provides structural support and promotes cell adhesion while silk fibroin offers biocompatibility and mechanical strength. The scaffold was implanted into rats with complete spinal cord injuries, and the results demonstrated that the combination of the scaffold and hUC-MSCs facilitated neural tissue regeneration and improved motor function recovery [41]. Wang et al. developed a nanofibrous scaffold using silk fibroin to support bone marrow stromal cells (BMSCs) for spinal cord injury repair. The scaffold was designed to mimic the natural extracellular matrix, providing a conducive environment for BMSC attachment and growth. In vivo experiments demonstrated that the implantation of this scaffold seeded with BMSCs facilitated neural tissue regeneration and improved motor function recovery in animal models [42] (Table 1).

Agarose is a linear polysaccharide that comes from the red algae Gelidium and Gracilaria. Its unique physicochemical properties make it a useful scaffold material for repairing SCI. Agarose is made up of repeating units of agarobiose. It can go from a sol to a gel when cooled below 35–40 °C without the use of chemical crosslinkers. This property makes it possible to deliver the substance as a liquid that solidifies on site and fits the shape of the injury cavity with little damage. When agarose gels, it makes a nanofibrous, highly hydrated, and mechanically strong hydrogel matrix with adjustable porosity. This matrix provides mechanical support that is essential for keeping the structure of the lesion cavity and stopping glial scar compaction. Agarose is biologically bioinert, which means it does not stick to proteins or cells in a non-specific way. This can help reduce inflammatory responses. This bioinertness, on the other hand, limits interactions between cells, so chemicals must be added to help neurons stick to each other, penetrate axons, and integrate with neuroglia. Researchers have made agarose-based scaffolds into multichannel structures that look like white matter tracts and help axons grow back across the lesion.

A flexible class of biomaterials extensively used in neural tissue engineering and SCI repair are gelatin and its GelMA derivative. Their adjustable rates of degradation; matrix metalloproteinase (MMP) sensitivity; and flexible crosslinking techniques, including UV-induced photopolymerization and enzymatic gelation, allow exact control over scaffold architecture and therapeutic release profiles. Designed as injectable scaffolds able of delivering growth factors, stem cells, or neuroprotective agents directly to the lesion site, GelMA hydrogels can be engineered to promote localized regeneration and minimize systemic toxicity. Crucially for neural repair, their natural bioactivity and ECM mimicry support cell adhesion, proliferation, and differentiation. Consistent clinical performance depends on addressing issues including thermosensitivity, batch-to-batch variability, and the necessity of chemical modification to enhance mechanical and degradation characteristics, however. Because of their adaptability and biocompatibility, gelatin-based hydrogels continue to be front and foremost in SCI treatment approaches, despite these restrictions [12,13].

Each biopolymer offers distinct advantages and challenges, influencing its applicability in hydrogel-based particle systems. Chitosan and alginate are highly effective for antimicrobial and drug delivery applications due to their ability to form gels through ionic gelation, while HA excels in tissue engineering and inflammation modulation. Silk fibroin, with its superior mechanical strength and functionalization potential, is ideal for neural tissue engineering. From a production perspective, ionic gelation methods in chitosan and alginate hydrogels provide simplicity and efficiency, whereas covalent crosslinking in HA and silk fibroin hydrogels offers greater tunability but often involves more complex procedures. By leveraging the complementary strengths of these biopolymers and addressing their individual limitations, bioactive hydrogels can be tailored to meet the multifaceted demands of advanced biomedical applications. Future research should focus on hybrid systems that combine multiple biopolymers to achieve synergistic effects, enabling personalized and highly effective therapies for spinal cord injuries, cancer, and infection control.

### Gelatin-Based Hydrogels in SCI Repair

Gelatin, a denatured derivative of collagen, has emerged as a foundational material in hydrogel design for SCI repair due to its intrinsic biocompatibility, bioactivity, and enzymatic degradability. Gelatin contains Arg-Gly-Asp (RGD) sequences that facilitate cell adhesion and supports neural cell viability, making it highly suitable for applications in neural tissue engineering [43]. Its methacrylated derivative, GelMA, offers enhanced control over physicochemical properties through photopolymerizable crosslinking, enabling precise tuning of mechanical strength, degradation rates, and porosity [44].

**Figure 2:**
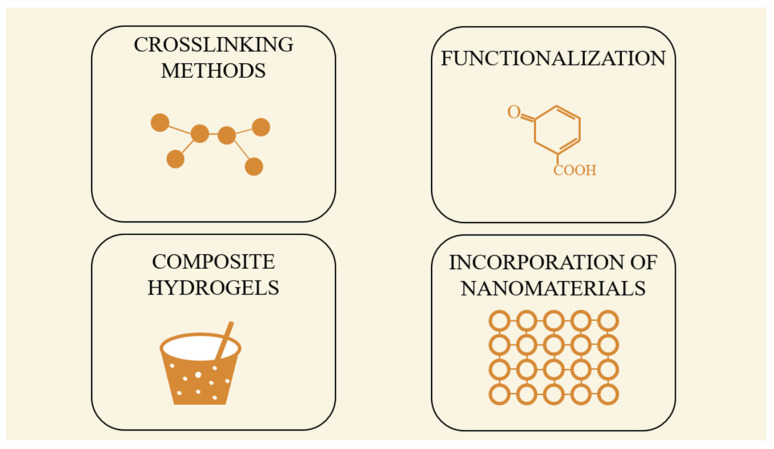
Advanced strategies to improve gelatin-based hydrogels.

The functionalization of gelatin with methacrylic anhydride to form GelMA allows for photo-crosslinking using UV or visible light, a process that retains cell viability and facilitates the encapsulation of sensitive bioactive agents. Additionally, enzymatic and thermal gelation methods enable the fabrication of gelatin-based scaffolds under physiological conditions. These approaches make gelatin hydrogels suitable for injectable therapies, minimizing surgical invasiveness. GelMA can also be incorporated into interpenetrating polymer networks (IPNs) with hyaluronic acid, alginate, or PEG to improve mechanical properties and longevity in vivo (Figure 2) [45].

Compared to other biopolymers like chitosan or alginate, gelatin-based hydrogels exhibit superior cell adhesion, tissue integration, and biodegradability compatible with the SCI healing timeline. Unlike alginate, which lacks inherent cell-binding domains, gelatin supports cellular attachment and migration. Moreover, its matrix metalloproteinase (MMP)-sensitive domains enable context-dependent degradation that mirrors native ECM remodeling, offering a regenerative advantage [45].

The versatility of gelatin has led to its adoption in several preclinical and translational models of SCI. For instance, Chen et al. developed GelMA hydrogel microspheres loaded with basic fibroblast growth factor (bFGF) for spinal nerve regeneration. These microspheres provided sustained bFGF release, supported neural progenitor cell proliferation, and facilitated axonal regrowth in SCI models, demonstrating promising functional recovery outcomes [13].

In another study, Liu et al. engineered a CeNP-GelMA hydrogel, incorporating cerium oxide nanoparticles with reactive oxygen species (ROS)-scavenging capacity. This hydrogel protected neural stem cells from oxidative stress, enhanced neuronal differentiation, and improved motor function recovery in SCI animal models, showcasing the potential of GelMA as a bioactive cell niche [46].

The continued refinement of GelMA synthesis, crosslinking chemistry, and functional nanoparticle integration has elevated gelatin-based hydrogels as one of the most promising scaffold systems for SCI repair. Their adaptability to 3D bioprinting, drug/cell delivery, and bioelectronic integration underscores their translational potential. Future work should focus on scaling GMP-compliant production, optimizing degradation profiles, and advancing clinical trial readiness through standardized preclinical protocols.

In comparison to its unmodified precursor, GelMA exhibits superior biochemical functionality and mechanical tunability, rendering it highly advantageous for SCI repair applications. Native gelatin, while inherently biocompatible and rich in cell-interactive motifs such as arginine-glycine-aspartic acid (RGD), suffers from significant limitations, including thermally labile gelation behavior and a lack of mechanical robustness. These constraints hinder its structural stability and restrict its utility in advanced tissue engineering strategies.

GelMA circumvents these issues through the covalent incorporation of methacryloyl groups onto gelatin’s amino acid side chains, thereby enabling photoinitiated crosslinking under ultraviolet or visible light in the presence of suitable photoinitiators. This modification imparts enhanced mechanical strength, spatial resolution in scaffold fabrication, and controlled degradation kinetics. The resulting hydrogel network preserves native bioactive motifs while allowing for precise architectural modulation via techniques such as 3D bioprinting and micropatterning. This enables the fabrication of biomimetic scaffolds with anisotropic properties and region-specific cue presentation, both of which are critical for guiding axonal regrowth and orchestrating neuroglial interactions within the injured spinal cord milieu.

Expanding the material landscape, other biopolymers have also demonstrated translational relevance in SCI repair. Agarose, a linear polysaccharide derived from red algae, is particularly notable for its high gel stiffness, thermal reversibility, and immunological inertness. Recent studies have shown that agarose-based scaffolds support axonal regeneration by forming a permissive, physically stable matrix that limits inflammatory cell infiltration and promotes neuronal extension across lesion sites [47,48,49].

Complementary to this, other naturally derived polymers such as chitosan and hyaluronic acid exhibit favorable biological profiles, including immunomodulatory and neuroprotective properties, making them suitable candidates for modulating the hostile post-injury microenvironment. Synthetic polymers like polyethylene glycol (PEG) derivatives, although lacking inherent bioactivity, provide high degrees of customization in terms of molecular weight, crosslinking density, and functionalization, allowing for tailored hydrogel systems with minimized immunogenicity and predictable in vivo behavior (Table 2).

## 4. Applications of Bioactive Hydrogels in SCI Repair

**Figure 3:**
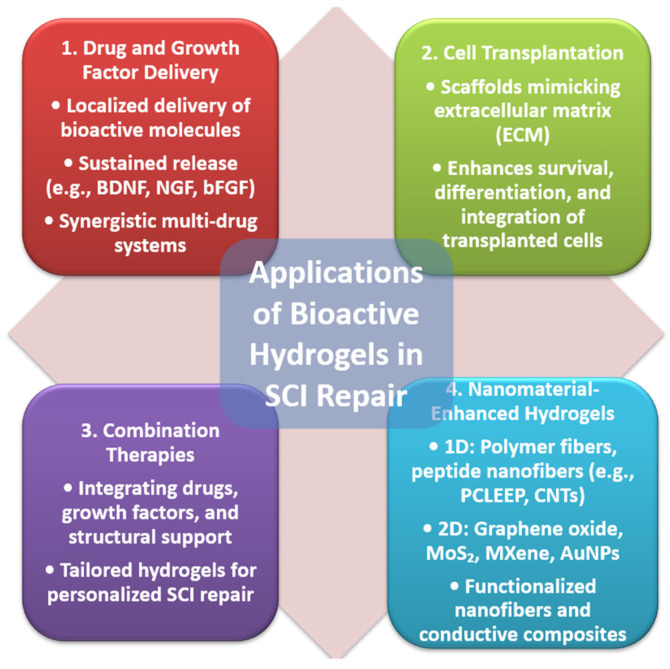
Bioactive hydrogels in SCI repair.

Bioactive hydrogels hold immense potential in the treatment of SCI due to their ability to serve as multifunctional platforms for delivering therapeutic agents, cells, and combinations of treatments. Their versatility enables precise targeting of the injury site, making them integral to advancing SCI repair strategies (Figure 3).

### 4.1. Drug and Growth Factor Delivery

Hydrogels are highly effective carriers for localized delivery of drugs and growth factors (GFs) in SCI repair. Their porous structure enables the encapsulation and sustained release of bioactive molecules, ensuring prolonged therapeutic effects while minimizing systemic side effects. By creating a controlled microenvironment at the injury site, hydrogels support anti-inflammatory interventions, axonal regeneration, and angiogenesis, making them invaluable in SCI treatment.

Injectable hydrogels, acting as 3D scaffolds, provide a conducive environment for drug and GF delivery. For example, Hassannejad et al. developed an amphiphilic peptide hydrogel for brain-derived neurotrophic factor (BDNF) delivery. This hydrogel achieved a controlled, slow release of BDNF over 21 days, retaining its biological activity while promoting neurite outgrowth, attenuating inflammation, and enhancing axonal preservation [53]. Similarly, Alizadeh and colleagues utilized a chitosan/β-glycerophosphate/hydroxyethyl cellulose (CTS/β-GP/HEC) hydrogel encapsulating mesenchymal stem cells (hADSCs) overexpressing nerve growth factor (NGF). This hydrogel effectively improved motor function recovery by promoting NGF expression and providing a supportive environment for cell survival and proliferation [54].

Hydrogels have also been used to deliver multiple agents simultaneously. Nazemi et al. constructed a dual-drug delivery hydrogel containing polylactic acid–glycolic acid microspheres for sustained release of nerve regeneration drug PTX and neuroprotective agent minocycline hydrochloride (MH). This system reduced inflammation, enhanced neuronal regeneration, and minimized scar tissue formation over eight weeks [55]. In a similar approach, Qi et al. combined cetuximab and FTY720 with neural stem cells (NSCs) in an injectable hydrogel, synergistically promoting neuronal differentiation, reducing bruising, and reconstructing nerve fiber networks [56].

In addition to GFs, hydrogels facilitate targeted drug delivery to improve therapeutic outcomes. For instance, Wang et al. reported a multi-drug delivery system using docetaxel (DTX) and acidic fibroblast growth factor (aFGF), encapsulated in a heparin-modified poloxamer hydrogel. This hydrogel formed a 3D network via self-assembly, enabling controlled drug release, enhanced drug penetration across the blood–spinal cord barrier, and local ECM reconstruction, thereby accelerating axonal regeneration and restoring signal conduction [57].

The use of hydrogels as delivery platforms extends to natural materials. Xu et al. developed decellularized spinal-cord-derived matrix (DSCM) hydrogels, which mimic the ECM’s nanofibrous structure, promoting neural stem/progenitor cell proliferation and differentiation [58]. In non-human primate models, Rao et al. used a chitosan hydrogel loaded with neurotrophic factor 3 (NT3), achieving slow, sustained GF release; robust axonal regeneration; and functional recovery [59].

Recent studies have explored innovative delivery systems for basic fibroblast growth factor (bFGF) to enhance its therapeutic potential in spinal cord and nerve injury repair. Wu et al. developed a barrier-penetrating liposome-based system for targeted delivery of bFGF to SCI sites. These liposomes demonstrated enhanced permeability and retention, effectively transporting bFGF across physiological barriers, leading to significant improvements in tissue repair and functional recovery in SCI models [60].

Chen et al. focused on GelMA hydrogel microspheres loaded with bFGF, designed for sustained release and spinal nerve regeneration. These microspheres exhibited controlled release kinetics, supported cell proliferation and differentiation, and facilitated spinal nerve repair [13]. Both approaches underscore the potential of advanced delivery systems, such as liposomes and hydrogel microspheres, in overcoming traditional challenges associated with bFGF therapy, paving the way for improved outcomes in SCI and nerve regeneration treatments.

A study by Ji et al. demonstrated that gelatin-based hydrogels crosslinked with genipin can effectively deliver basic fibroblast growth factor (bFGF) for spinal cord injury repair. By adjusting genipin concentrations, they tuned the hydrogel’s mechanical properties and degradation rate. In vitro tests confirmed sustained bFGF release and Schwann cell viability. In vivo, implantation into rat spinal cord hemisection models showed that 0.5% genipin-crosslinked hydrogels enhanced tissue regeneration and biocompatibility [61]. Another study by Furuya et al. investigated the effects of basic fibroblast growth factor (bFGF)-incorporated gelatin hydrogel (GH) on spinal cord injury in rats. GH was used as the delivery scaffold, and 20 µg of bFGF was embedded and injected into the lesion site one week post-contusion. Although no significant improvements in locomotor function were observed, the bFGF–GH group exhibited reduced mechanical allodynia compared to controls, suggesting potential safety and benefit for alleviating sensory abnormalities post-SCI [62]. A study by Shen et al. designed an injectable, thermosensitive hydrogel composed of β-cyclodextrin-modified hyaluronic acid, gelatin, and poloxamer (CD-HA/Gel/Poloxamer) for the co-delivery of methylprednisolone (MP) and nerve growth factor (NGF) in SCI treatment. The hydrogel exhibited sol–gel transition at physiological temperature and provided sustained, sequential release of MP and NGF, enhancing drug stability and bioavailability. In vitro, the system showed favorable biocompatibility and anti-inflammatory properties. In vivo, the hydrogel was administered at the injury site in a rat SCI model, resulting in reduced inflammation, suppressed glial scar formation, and improved axonal regeneration and functional recovery. These findings demonstrate the potential of CD-HA/Gel-based hydrogels as multifunctional platforms for synergistic drug and growth factor delivery in SCI therapy [63].

The ability of hydrogels to deliver therapeutic agents precisely and sustainably enhances their value in SCI repair. Future innovations in hydrogel design will likely focus on improving specificity, biocompatibility, and the synergistic effects of combined therapies, offering new hope for effective SCI treatments.

### 4.2. Cell Transplantation

Hydrogels play a crucial role as supportive scaffolds for cell transplantation in SCI repair by providing a microenvironment that mimics the extracellular matrix (ECM). This microenvironment enhances the survival, differentiation, and integration of encapsulated cells, such as stem cells, Schwann cells, and neural progenitor cells, at the injury site [64]. Hydrogels also promote cell migration and organization, which are essential for bridging damaged tissue, remyelination, and axonal guidance. Advanced hydrogels are often functionalized with bioactive cues, such as cell-adhesive peptides, to further optimize cell behavior and improve therapeutic outcomes [65].

To combat the challenges posed by reactive oxygen species (ROS) during SCI repair, functional nanoparticles can be incorporated into hydrogels to scavenge ROS and promote cell survival. For instance, Liu et al. synthesized CeNP-Gel hydrogels by dispersing cerium oxide nanoparticles (CeNPs) with enzyme-like ROS-scavenging activity into GelMA. These hydrogels significantly enhanced the survival and differentiation of neural stem cells (NSCs) by reducing oxidative damage [46].

Similarly, MnO_2_ nanoparticles (NPs) have been integrated into hydrogels to catalyze the decomposition of ROS, such as H_2_O_2_, into harmless by-products. Li et al. developed MnO_2_-embedded hydrogels using a peptide-modified hyaluronic acid (HA) matrix. These hydrogels promoted nerve fiber regeneration and inhibited glial scar formation, which was confirmed by reduced levels of glial fibrillary acidic protein (GFAP)-positive astrocytes and improved angiogenesis [66].

Conductive hydrogels are another innovative approach in SCI repair, facilitating electrical signal transmission to enhance neural tissue regeneration. Wu et al. prepared conductive nanohydrogels by incorporating polypyrrole (PPy) nanoparticles into a HA-collagen matrix. These hydrogels not only protected bone marrow stem cells (BMSCs) from oxidative damage but also promoted their neuronal differentiation through electrical conductivity and activation of signaling pathways like PI3K/Akt and MAPK [67].

Magnetoelectric properties have also been utilized to enhance SCI repair. Zhang et al. decorated HA-collagen hydrogels with Fe_3_O_4_@BaTiO_3_ nanoparticles, enabling the hybrid hydrogels to support spinal nerve repair under a pulsed magnetic field [68]. Ko et al. explored a thermogenic approach by incorporating gold nanoparticles (AuNPs) conjugated with ursodeoxycholic acid (UDCA) into a chitosan-HA hydrogel. Upon near-infrared (NIR) irradiation, this hydrogel generated localized heat, reduced inflammatory cytokine production, and created an anti-inflammatory environment for effective SCI repair [69].

Through their ability to mimic the ECM, modulate inflammation, and integrate functional nanoparticles, hydrogels offer a powerful platform for enhancing the effectiveness of cell transplantation in SCI repair. Continued innovations in hydrogel design will likely expand their clinical potential and improve outcomes for patients with SCI.

### 4.3. Combination Therapies

The multifaceted nature of SCI repair often necessitates combination therapies, and hydrogels provide a unique platform for integrating multiple treatment modalities. By simultaneously delivering drugs, growth factors, and cells, hydrogels address complex challenges such as inflammation, neural regeneration, and vascularization while providing mechanical support and mimicking the extracellular matrix (ECM). The ability to tailor hydrogels for specific treatments enables a synergistic approach to SCI repair and offers great promise for personalized therapies.

One-dimensional material (1DM)-based hydrogels, composed of nanoscale materials with one-dimensional morphologies such as polymer fibers, carbon nanotubes (CNTs), and peptide nanofibers, have shown remarkable potential in SCI repair. These materials enhance the mechanical properties and functionality of hydrogels, making them ideal for combination therapies. For instance, Nguyen et al. developed aligned poly(ε-caprolactone-co-ethyl ethylene phosphate) (PCLEEP) nanofibers embedded in a collagen matrix. This biomimetic 3D architecture provided a versatile platform for delivering drugs and genes like neurotrophin-3 and microRNA, promoting robust axonal regeneration, cell adhesion, and neurite infiltration [70]. Similarly, Li et al. fabricated a hybrid hyaluronic acid (HA)-based hydrogel doped with electrospun polycaprolactone (PCL) fibers. The hydrogel exhibited mechanical properties similar to native spinal cord tissue while effectively supporting axonal growth, neuro-regeneration, and macrophage polarization [71] (Table 3).

CNTs have been utilized to enhance the conductivity and mechanical strength of hydrogels, promoting electrical signal transmission and nerve regeneration. Liu et al. developed electrically conductive nanocomposite hydrogels embedded with functional nanoparticles for SCI repair. These hydrogels were designed to mimic the natural extracellular matrix while providing electrical conductivity to enhance neuronal signaling. By incorporating nanoparticles, the hydrogels exhibited improved mechanical strength, biocompatibility, and electrical properties, which collectively supported axonal regrowth and neural tissue repair. The study highlights the potential of electrically conductive hydrogels as a multifunctional platform, combining structural support with bioelectrical modulation, to promote functional recovery following SCI [72].

Two-dimensional materials (2DMs), such as graphene oxide (GO), reduced graphene oxide (rGO), molybdenum disulfide (MoS_2_), and MXene, have also been incorporated into hydrogels to provide unique properties such as high conductivity, mechanical strength, and biocompatibility. Girão et al. highlighted the promise of graphene-based hydrogels in SCI repair due to their ability to enhance neural cell–material interactions and promote neuroregeneration [73]. Chen et al. demonstrated a MoS_2_/GO hybrid hydrogel, which combined the conductivity of MoS_2_ with the mechanical strength of GO to facilitate neural stem cell (NSC) differentiation, reduce inflammation, and promote neural repair [74]. Similarly, Kong et al. incorporated MXene and gold nanoparticles (AuNPs) into GelMA hydrogels to create a conductive scaffold for NSC delivery, supporting myelin regeneration and functional recovery of SCI [75].

Aligned fibrin nanofibers (AFGs) and peptide nanofibers have been functionalized to enhance their bioactivity and regenerative potential in SCI repair. Yang et al. tailored AFGs with N-cadherin to improve central nervous system functions and axonal regeneration [76]. Similarly, silk fibroin nanofiber (SFN) hydrogels doped with nerve growth factor (NGF) have shown promising results in guiding scarless neural regeneration and restoring motor functions in animal models [77]. Composite hydrogels containing rGO and xanthan gum demonstrated enhanced electroconductivity, promoting nerve fiber regeneration and reducing glial scar formation [78]. Hydrogels provide an unparalleled platform for combination therapies, integrating drugs, growth factors, and structural support into a single system. The incorporation of 1DM and 2DM materials further enhances the functionality of hydrogels, enabling tailored treatments for complex SCI repair. By addressing the diverse needs of tissue regeneration, inflammation modulation, and neuroprotection, these advanced hydrogels hold immense potential for personalized and effective SCI therapies.

## 5. Analysis of Publication Trends

Over the past decade, there has been a notable increase in research output related to the use of hydrogels and biopolymers in SCI repair. A PubMed search revealed 121 free-text publications in the last 10 years, with 95 of these articles published in the last 5 years. This marked increase in recent publications underscores the growing interest and progress in exploring biomaterials for SCI treatment. Researchers have investigated a diverse range of approaches, including the synthesis and functionalization of hydrogels, the integration of biopolymers, and the development of advanced delivery systems. These efforts highlight continuous innovation and the dynamic nature of this field.

Despite this robust publication rate, a detailed analysis reveals an extremely low number of studies classified as “clinical trials” or “clinical studies”. This trend suggests a significant translational gap between preclinical research and clinical application. Several factors contribute to this disparity. Variability in experimental designs across studies, differences between preclinical models and human physiological conditions, and challenges in accurately replicating these conditions all hinder the direct translation of findings. Furthermore, the absence of standardized evaluation protocols and the inherent complexity of the SCI repair process exacerbate the difficulties in applying preclinical results to clinical settings. Addressing these challenges through more uniform methodologies and advanced modeling techniques will be essential for bridging the gap between preclinical advancements and clinical implementation, ultimately improving outcomes for SCI patients.

## 6. Costs of Hydrogel Therapy in Spinal Cord Injury: Current Trends and Historical Perspective

The cost of hydrogel therapy in SCI repair is influenced by various factors, including the raw materials used, production techniques, and advancements in technology. Over time, the costs associated with hydrogel production have undergone significant changes due to improvements in synthesis methods, the incorporation of advanced materials, and economies of scale [79]. Hydrogels utilized in SCI therapy often involve biopolymers such as hyaluronic acid, chitosan, and alginate, as well as synthetic polymers like polyethylene glycol (PEG) and polylactic acid-glycolic acid (PLGA) [80]. These materials vary widely in cost depending on their source, purity, and the modifications required to enhance their properties. Naturally derived polymers such as hyaluronic acid are typically more expensive than synthetic polymers due to the complex extraction and purification processes involved. The addition of nanoparticles, growth factors, or conductive materials like graphene oxide (GO) further increases production costs, reflecting the complexity and functionality of these advanced hydrogel systems [81].

Methods of production and crosslinking also significantly impact costs. Techniques such as ionic gelation, photopolymerization, and self-assembly require specialized equipment and expertise, which can increase expenses. However, advances in scalable production technologies and automation have helped reduce costs. For example, the use of 3D printing and biofabrication for hydrogel scaffolds has enabled precise control over structure and functionality while minimizing material wastage, contributing to cost efficiency [82].

Historically, hydrogel production for biomedical applications was more expensive due to the limited availability of raw materials, less efficient synthesis methods, and the absence of advanced functionalization techniques. Early hydrogel systems relied on basic polymers with minimal properties, which restricted their applicability and required significant downstream modifications to meet therapeutic needs. Functionalization with bioactive molecules and advanced materials, now common practice, was both technically challenging and prohibitively expensive in earlier years. Over the past decade, advancements in biopolymer processing, improvements in crosslinking methods, and increased availability of high-quality materials have contributed to significant cost reductions [26]. For instance, the production cost of alginate and chitosan-based hydrogels has decreased with improved industrial-scale extraction methods. Similarly, the widespread adoption of PEG and PLGA, coupled with progress in polymer chemistry, has made synthetic hydrogels more affordable and customizable [83].

Today, the cost of hydrogel production for SCI therapy is considerably lower than a decade ago, with average reductions of 30% to 50%, depending on the material and production method. For instance, processes involving the integration of nanoparticles, which were previously labor-intensive and expensive, have become more cost-effective due to scalable production techniques. Additionally, advancements in recombinant protein production and peptide synthesis have reduced the costs associated with functionalizing hydrogels with growth factors or bioactive peptides [84] (Table 4).

Despite these advancements, certain challenges remain. High-grade materials requiring extensive purification or complex functionalization continue to drive up costs. Regulatory requirements for medical-grade hydrogels further add to expenses due to the rigorous testing and quality assurance needed. Personalized hydrogel systems, designed to suit individual patients or specific injury types, introduce additional complexity and expense to the production process. While the costs of hydrogel therapy for SCI have decreased significantly, they remain a barrier to widespread clinical adoption. Addressing these challenges through further advancements in production techniques, material science, and regulatory processes will be critical to making hydrogel therapy more accessible. As these barriers are overcome, hydrogel-based treatments have the potential to become more broadly available, paving the way for improved clinical outcomes for SCI patients.

## 7. Conclusions and Future Perspectives

SCI continues to represent one of the most complex and debilitating conditions in medicine, often leading to irreversible loss of function and limited therapeutic options. In recent years, bioactive hydrogels have gained significant attention as a regenerative strategy, offering biomimetic environments that support cell viability, guide axonal growth, and facilitate the localized delivery of therapeutic agents. These materials have evolved to include advanced functionalities such as self-healing, electrical conductivity, and programmable biodegradation—each contributing to more effective modulation of the SCI microenvironment.

Despite these promising advances, the path toward clinical application remains hindered by several persistent challenges. One of the most pressing needs is the development of scalable, GMP-compliant manufacturing processes. Ensuring batch consistency, sterility, and regulatory compliance is crucial for transitioning hydrogel technologies from the lab bench to the clinic. Equally important is the adoption of standardized preclinical models and evaluation protocols that reflect clinically relevant endpoints.

Another emerging frontier lies in the integration of artificial intelligence and machine learning into hydrogel design. By leveraging predictive modeling and high-throughput data analysis, researchers can more efficiently optimize hydrogel formulations, anticipate in vivo responses, and tailor materials to specific injury profiles. This data-driven approach holds significant promise for accelerating discovery and reducing translational bottlenecks.

Furthermore, the convergence of 3D bioprinting with patient-specific anatomical data offers new opportunities for personalized hydrogel scaffolds. These customized constructs can be engineered to match the unique geometry and pathology of individual lesions, thereby improving graft-host integration and therapeutic efficacy.

As the field continues to mature, targeted efforts in these areas will be essential to unlocking the full potential of hydrogel-based therapies for SCI. By addressing the scientific, technological, and regulatory barriers that remain, bioactive hydrogels are well-positioned to transform the landscape of neuroregenerative medicine and offer renewed hope for patients living with spinal cord injuries.

## Figures and Tables

**Table 1:** Comparative analysis of biopolymers: properties, applications, and challenges.

Biopolymer	Key Properties	Applications	Challenges	Recent Innovations
Gelatin/GelMA	Tunable degradation rate, MMP-sensitive, versatile crosslinking (UV, enzymatic)	Injectable scaffolds, growth factor delivery, neural and spinal repair	Thermosensitivity, batch variability, requires chemical modification for stability	Three-dimensional bioprinting of structured GelMA networks; conductive GelMA for neural interfaces
Chitosan	Biocompatible, antimicrobial, forms gels under mild conditions, cationic nature	Encapsulation of nanoparticles, wound healing, drug delivery	Limited solubility at physiological pH, weak mechanical properties	Chitosan-based injectable nanogels; co-delivery systems with growth factors or EVs
Hyaluronic acid	Biocompatible, promotes cell adhesion and proliferation, modulates inflammation	Neural tissue engineering, cancer therapy, targeted delivery	Rapid degradation, limited mechanical strength	HA hydrogels with enzymatic resistance; HA-integrated stem cell scaffolds
Alginate	Biocompatible, easy gelation, porous structure for drug encapsulation	Sustained drug release, neuroprotection, anti-inflammatory use	Lacks intrinsic cell adhesion	Oxidized alginate for tunable degradation; RGD-functionalized alginate scaffolds
Silk fibroin	Strong mechanical properties, biocompatible, slow degradation	Neural tissue engineering, scaffolding	Complex processing, chemical modification required	Composite silk-based scaffolds; aligned silk fibers for axonal guidance
Agarose	Thermoreversible gelation, high mechanical stiffness, inert to cell signaling unless modified	Multichannel guidance conduits, axonal bridging matrices, structural supports in lesion cavities	Poor intrinsic bioactivity, limited cell adhesion, difficult to functionalize for specific cell types	Functionalized agarose with peptides or neurotrophins; multichannel agarose constructs

**Table 2:** Summary of biopolymers used in SCI repair [50,51,52].

Biopolymer	Crosslinking Method	Cell Compatibility	Mechanical Stability	Key Benefits	Potential Risks
Gelatin	None or enzymatic	Moderate	Low	Biocompatible, inexpensive	Poor mechanical properties
GelMA	Photoinitiated (UV/visible)	High	Tunable	Tailored stiffness, bioactive	Light/initiator toxicity
Agarose	Thermal gelation	High	High	Biostable, immuno-inert	Limited bioactivity
Chitosan	Ionic/enzymatic	High	Moderate	Anti-inflammatory, antimicrobial	Solubility and variability
Hyaluronic acid	Enzymatic/chemical	High	Tunable	Neuroprotective, ECM mimic	Rapid degradation
PEG derivatives	Click chemistry/photocrosslink	High	Precisely tunable	Predictable, customizable	Non-bioactive, synthetic

**Table 3:** One-dimensional and two-dimensional nanomaterial-enhanced hydrogels for SCI repair.

Material Type	Representative Materials	Hydrogel Integration	Key Benefits in SCI Repair	Example Studies
One-dimensional nanomaterials	Aligned polymer nanofibers (e.g., PCLEEP), electrospun PCL fibers, CNTs, peptide nanofibers	Embedded in collagen, HA, or GelMA matrices	Enhanced mechanical strength, neurite guidance, drug/gene delivery, electrical conductivity	[70,71,72]
Two-dimensional nanomaterials	Graphene oxide (GO), reduced GO (rGO), MoS_2_, MXene, AuNPs	Mixed with GelMA, composite networks	Improved conductivity, biocompatibility, NSC differentiation, inflammation control	[73,74,75]
Functionalized nanofibers	Aligned fibrin nanofibers (AFGs), silk fibroin nanofibers (SFNs)	Blended into hydrogels with growth factors like NGF	Enhanced axonal regeneration, scar-free healing, CNS function restoration	[76], SFN-NGF models
Composite platforms	rGO + xanthan gum, MoS_2_/GO hybrids, MXene + AuNPs	Conductive composite hydrogels	Electroconductivity, glial scar suppression, myelin regeneration	[74,75], AFG-tailored systems

**Table 4:** Cost comparisons and regulatory considerations [85,86,87].

Material	Estimated Cost (USD/mL)	Notes	Regulatory/Reimbursement Context
GelMA	USD 36–38/mL	Advanced BioMatrix 20% solution: USD 180 for 5 mL → USD 36/mL. CELLINK GelMA bioink: USD ≈ 38/mL.	Synthetic gelatin-based biomaterials often undergo GMP track; products for bioinks need sterility certification; reimbursement still limited due to classification as “experimental”.
Chitosan-based hydrogel	USD ≈4/mL (thermosensitive gelatin–chitosan)	Reported at USD ~4/mL in PLoS One study. Prices may increase by 2–3× when functionalized with growth factors or in high-purity medical grades. Chitosan hydrogels occasionally gain medical device approval; some wound-care chitosan hydrogels already reimbursed under skin grafts or adhesives.	
Hyaluronic acid (HA)-based cHydrogel	USD 30–50/mL	Market bioinks like PhotoHA-INK cost USD 325 for 5 mL → USD 65/mL; actual therapeutic-grade hydrogels run USD ~30–50/mL.	HA is widely FDA-approved for fillers and joint injections; high-ni HT-grade therapeutic hydrogels still under OUS or clinical trials.

## Data Availability

No new data were created or analyzed in this study.
